# The effect of celecoxib on tumor growth in ovarian cancer cells and a genetically engineered mouse model of serous ovarian cancer

**DOI:** 10.18632/oncotarget.8659

**Published:** 2016-04-08

**Authors:** Anuj Suri, Xiugui Sheng, Kevin M. Schuler, Yan Zhong, Xiaoyun Han, Hannah M. Jones, Paola A. Gehrig, Chunxiao Zhou, Victoria L. Bae-Jump

**Affiliations:** ^1^ Houston Methodist Gynecologic Oncology Associates, Houston, TX, USA; ^2^ Department of Gynecologic Oncology, Shandong Cancer Hospital & Institute, Jinan University, Jinan, Shandong, China; ^3^ Good Samaritan Hospital, Division of Gynecologic Oncology, Cincinnati, OH, USA; ^4^ Division of Gynecological Oncology, University of North Carolina at Chapel Hill, Chapel Hill, NC, USA; ^5^ Department of Gynecologic Oncology, Linyi Cancer Hospital, Shandong, China; ^6^ Lineberger Comprehensive Cancer Center, University of North Carolina at Chapel Hill, Chapel Hill, NC, USA

**Keywords:** ovarian cancer, COX-2 inhibitor, celecoxib, obesity, genetically engineered mouse model

## Abstract

Our objective was to evaluate the effect of the COX-2 inhibitor, celecoxib, on (1) proliferation and apoptosis in human ovarian cancer cell lines and primary cultures of ovarian cancer cells, and (2) inhibition of tumor growth in a genetically engineered mouse model of serous ovarian cancer under obese and non-obese conditions. Celecoxib inhibited cell proliferation in three ovarian cancer cell lines and five primary cultures of human ovarian cancer after 72 hours of exposure. Treatment with celecoxib resulted in G1 cell cycle arrest, induction of apoptosis, inhibition of cellular adhesion and invasion and reduction of expression of hTERT mRNA and COX-2 protein in all of the ovarian cancer cell lines. In the KpB mice fed a high fat diet (obese) and treated with celecoxib, tumor weight decreased by 66% when compared with control animals. Among KpB mice fed a low fat diet (non-obese), tumor weight decreased by 46% after treatment with celecoxib. In the ovarian tumors from obese and non-obese KpB mice, treatment with celecoxib as compared to control resulted in decreased proliferation, increased apoptosis and reduced COX-2 and MMP9 protein expression, as assessed by immunohistochemistry. Celecoxib strongly decreased the serum level of VEGF and blood vessel density in the tumors from the KpB ovarian cancer mouse model under obese and non-obese conditions. This work suggests that celecoxib may be a novel chemotherapeutic agent for ovarian cancer prevention and treatment and be potentially beneficial in both obese and non-obese women.

## INTRODUCTION

Epithelial ovarian cancer (OC) is one of the most deadly cancers with an overall 5-year survival of only 30-40% [1, 2]. Although most OC patients initially respond to surgical debulking followed by carboplatin/taxane chemotherapy, the vast majority of women will recur and succumb to their disease. More than 21,000 cases are diagnosed annually in the US, and more than 14,000 women die in the same period [3]. Increasing evidence suggests that obesity is a significant risk factor for OC and is associated with worse outcomes for this disease [4–18]. Therefore, a metabolic approach to the treatment of OC may provide a novel strategy to improve outcomes for this invariably lethal disease.

Obesity induced inflammation leads to the induction of cyclooxygenase-2 (COX-2) expression and subsequent prostaglandin (PG) and pro-inflammatory cytokines production, which may directly favor ovarian carcinogenesis. COX-2, the rate-limiting enzyme in prostaglandin synthesis, has been found to be overexpressed and associated with poor prognosis in a number of different cancers, including ovarian cancers [19]. COX-2 has been shown to affect tumor progression through the inhibition of apoptosis and the promotion of proliferation, angiogenesis, and invasion [20, 21]. Celecoxib is a selective COX-2 inhibitor in the NSAID class which is primarily used as an anti-inflammatory drug in the treatment of conditions such as rheumatoid arthritis, osteoarthritis or severe cases of dysmenorrhea [20–23]. Epidemiological studies have indicated that long term use of celecoxib may reduce the risk of carcinogenesis in breast, prostate, lung and liver cancer [20–23]. Celecoxib has been shown to have anti-proliferative and anti-tumorigenic effects *in vitro* and *in vivo* for a number of different cancers [20, 21]. Thus, our objective was to evaluate the effect of celecoxib, on (1) proliferation and apoptosis in ovarian cancer cell lines and primary cultures of ovarian cancer cells, and (2) inhibition of tumor growth in a genetically engineered mouse model of serous ovarian cancer under obese and non-obese conditions.

## RESULTS

### Effect of celecoxib on ovarian cancer cell proliferation, COX-2 expression and PEG2 production

**Figure 1:**
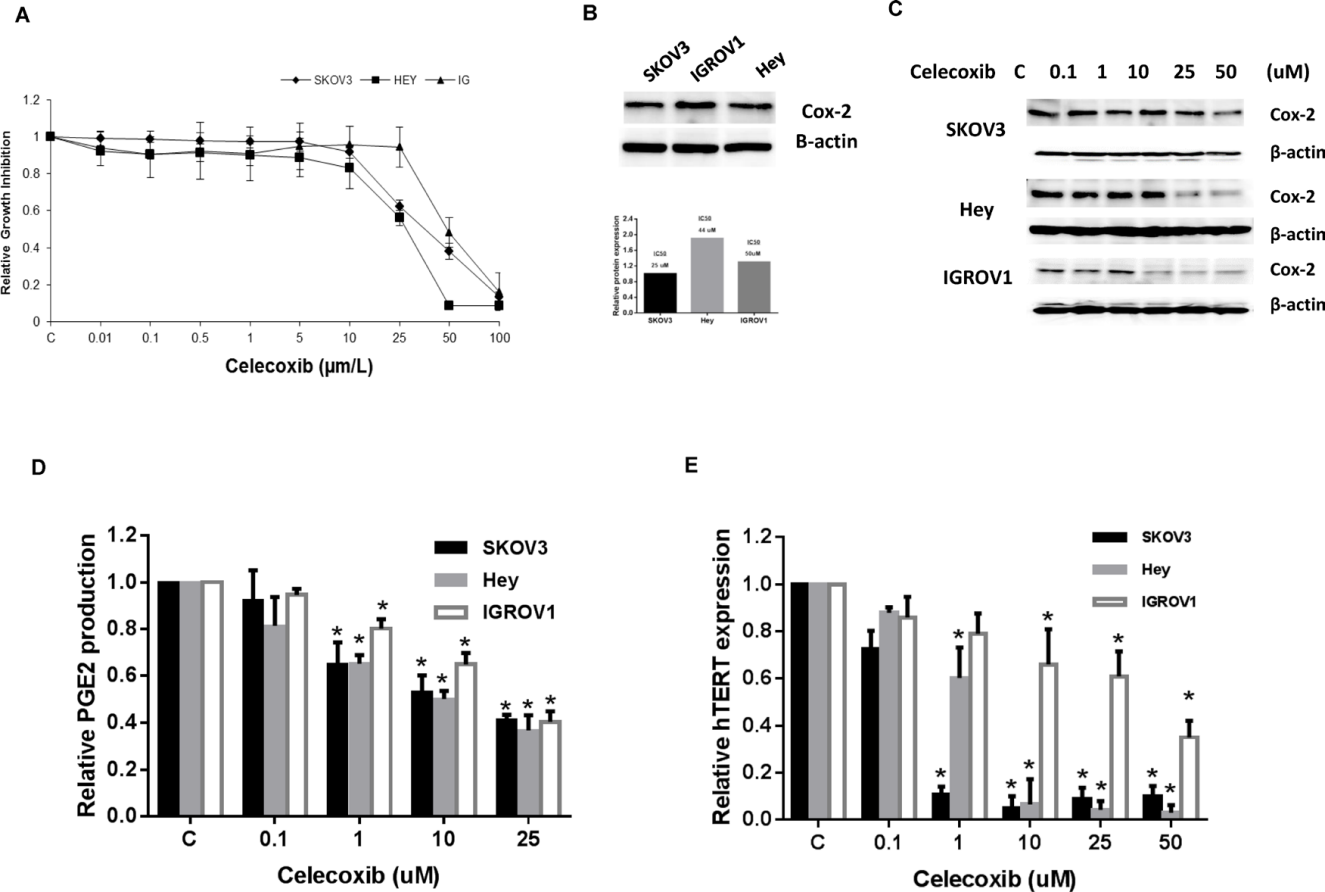
Celecoxib inhibited cell proliferation in ovarian cancer cell lines The SKOV3, Hey and IGROV1 cells were cultured for 24 hours and then treated with celecoxib at indicated doses in 96 well plates for 72 hours. Cell proliferation was assessed by MTT assay **A.** The three ovarian cancer cell lines have varying levels of COX-2 protein expression, and the sensitivity to celecoxib do not relate to protein expression of COX-2 **B.** Western blotting results indicated that celecoxib inhibited COX-2 protein expression in a dose-dependent manner after 24 hours treatment **C.** Celecoxib decreased PGE2 production in the media in ovarian cancer cells after 18 hours treatment **D.** Real time PCR results showed that celecoxib reduced hTERT mRNA expression after 24 hours treatment **E.** (* < 0.05).

The effect of celecoxib on ovarian cancer cell proliferation was assessed by MTT assay. As shown in Figure 1A, celecoxib inhibited cell growth in the three ovarian cancer cell lines in a dose dependent manner after 72 hours of exposure. The mean IC50 value for SKOV3, HEY and IGROV1 was 25, 44 and 50 uM (p = 0.0001-0.0002), respectively.

All three ovarian cancer cell lines expressed COX-2 (Figure 1B). Celecoxib significantly inhibited COX-2 protein expression in a dose dependent manner in all three ovarian cancer cell lines, as demonstrated by Western immunoblotting (Figure 1C). In addition, celecoxib (1-25 μM) significantly suppressed PEG2 production in the media in all three ovarian cancer cells after 18 hours of exposure (Figure 1D) (p < 0.05), as assessed by ELISA assay. Given that hTERT expression is thought to be a sensitive marker of telomerase function as well as cell proliferation, we next measured hTERT mRNA expression in our three ovarian cancer cell lines by real-time RT-PCR. Treatment with celecoxib at varying concentrations (1 – 50 μM) for 24 hours significantly decreased hTERT mRNA expression in a dose-dependent manner in the ovarian cancer cell lines (Figure 1E) (p < 0.05).

### Celecoxib induces cell cycle arrest in G0/G1 and apoptosis

**Figure 2:**
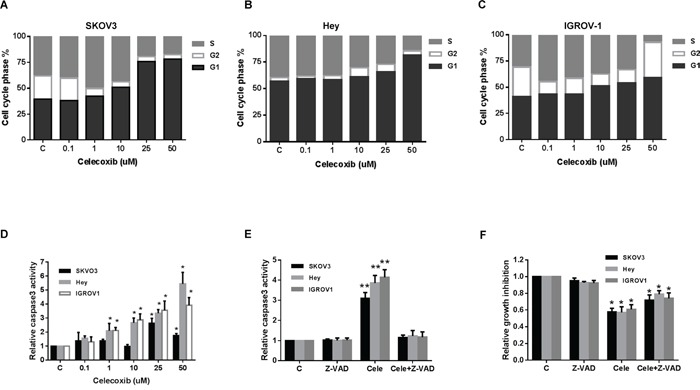
Celecoxib induced cell cycle G1 arrest and apoptosis in ovarian cancer cells lines The SKOV3, Hey and IGROV1 cells were treated with celecoxib at the indicated dose for 24 hours and then analyzed for cell cycle distributions by flow cytometry. Cell cycle phase analysis is shown in SKOV3, Hey and IGROV1 as representative of one of three independent experiments **A-C.** Celecoxib induced the activity of cleave caspase-3 in the ovarian cancer cell lines after 16 hours of treatment **D.** Pretreatment with a pan-caspase inhibitor (Z-VAD-FMK) at 20 um for 2 hours totally blocks cleaved caspase 3 activity induced by celecoxib **E.** Blocking caspase activity with Z-VAD-FMK resulted in a decrease in celecoxib-mediated growth inhibition in the ovarian cancer cell lines **F.** (* < 0.05).

To evaluate the underlying mechanism of growth inhibition by celecoxib, the cell cycle profile was analyzed after treating the SKOV3, Hey and IGROV1 cell lines with varying doses of celecoxib (0.1-50 uM) for 24 hours. As shown in Figure 2A–2C, celecoxib induced G0/G1 cell cycle arrest and reduced S phase in a dose-dependent manner in the ovarian cancer cell lines. Caspases play a central role in the induction of apoptosis. Caspase-3 is a member of the caspase family, which consists of cysteine proteases that act in a cascade manner to trigger apoptosis, and is considered to be one of the effector caspases involved in cell disassembly [24]. To determine whether caspases were involved in celecoxib-induced apoptosis in the ovarian cancer cell lines, cleaved caspase-3 activity was determined in the SKOV3, Hey and IGROV1 cell lines after treatment with celecoxib for 16 hours. As shown in Figure 2D, treatment with celecoxib (0.1–50 μM) significantly induced caspase-3 activity by 1.7, 5.4 and 3.8 fold at a dose of 50 uM compared to control in the SKOV3, Hey and IGROV1 cells (p < 0.05). These results suggest that celecoxib reduces cell proliferation through both the induction of cell cycle G1 arrest and apoptosis in ovarian cancer cells.

To further assess the possible role of apoptosis in celecoxib-treated ovarian cancer cells, we used the pan-caspase inhibitor (Z-VAD-FMK) to block caspase activity along with celecoxib treatment. Cells were pretreated with Z-VAD-FAM at 20 um for 2 hours before treatment of celecoxib at 1 um for 16 hours. The results showed that pre-treatment with Z-VAD-FMK total blocked the caspase 3 activity induced by celecoxib (Figure 2E). Furthermore, blocking caspase activation resulted in a significant decrease in celecoxib-mediated growth inhibition in all three ovarian cancer cell lines after 72 hours of treatment with celecoxib, suggesting that apoptosis may be a major mechanism for the inhibition of cell proliferation seen in celecoxib treated ovarian cancer cells (Figure 2F).

### Effect of celecoxib on adhesion and invasion in ovarian cancer cells

**Figure 3:**
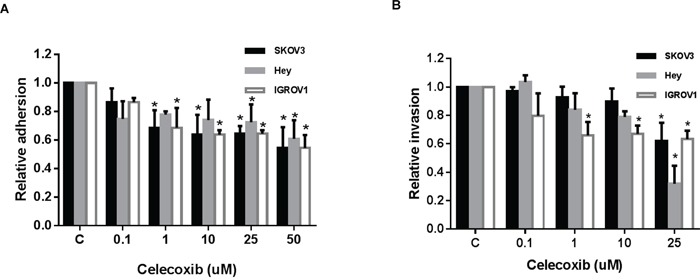
Celecoxib inhibited cell adhesion and invasion in ovarian cancer cells lines The ovarian cancer cells were treated with celecoxib at a range of dose from 0.1 −1 50 μM. Adhesion was assessed by laminin-1 assay after 2 hours of treatment with celecoxib **A.** Invasion was determined by ChemoTx invasion kit, after 4 hours of treatment with celecoxib **B.** (* < 0.05).

To analyze the effect of celecoxib on adhesion and invasion in ovarian cancer cells, an *in vitro* laminin adhesion assay and ChemoTx invasion system were utilized, respectively. Cellular adhesion was decreased by 20-40% in all three ovarian cancer cell lines at a dose of 25 mM (p = 0.002-0.009) and at 50 mM (p = 0.00001-0.02), after 2 hours of treatment (Figure 3A). Three ovarian cancer cells treated with celecoxib at a dose 25 uM for 4 hours resulted in a significantly decreased invasive ability of 39-69% (p<0.001) (Figure 3B). These results suggest that celecoxib reduces adhesion and invasion of ovarian cancer cells.

### Celecoxib inhibits tumor growth in the KpB serous ovarian cancer mouse model

The KpB mice began the LFD (Low fat diet) or HFD (High fat diet) at 3 weeks of age and were divided into four groups (N = 15 mice per group), including LFD (non-obese) and HFD (obese) groups treated with either celecoxib or placebo. The initial average body weight of the obese mice when starting treatment with celecoxib (5 mg/kg, 4 weeks) was 48.4 gm, while that of the non-obese mice was only 29.78 gm (p<0.01, data not shown).

**Figure 4:**
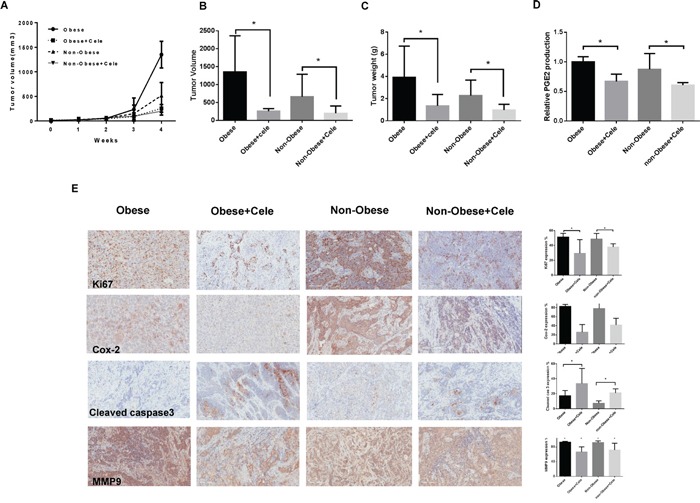
Celecoxib inhibited tumor growth in a genetically engineered mouse model of serous ovarian cancer (KpB mouse model) The KpB mice were fed a HFD (obese) or LFD (non-obese) starting at 3 weeks of age. Once a 0.1×0.1 cm ovarian tumor was palpated, these mice underwent treatment with celecoxib (5 mg/kg) versus placebo for 4 weeks. Celecoxib significantly inhibited tumor volume and tumor weight **A, B, and C.** Celecoxib reduced the level of serum PGE2 in the HFD- and LFD-fed groups **D.** The effect of celecoxib on the cell proliferation index, COX-2, apoptosis and invasion was assessed through expression of Ki67, COX-2, cleaved caspase3, and MMP9, respectively, as evaluated through immunohistochemistry **E.** (* p< 0.05).

Regular twice-weekly measurements yielded no changes in blood glucose or weight (data not shown) during celecoxib or placebo treatment. A substantial reduction in tumor growth and tumor weight was found in the celecoxib group in comparison with the placebo group (Figure 4A–4C). In the obese KpB mice, tumor weight decreased by 66% (p<0.05) with celecoxib treatment when compared with control-treated animals. Among non-obese KpB mice, tumor weight decreased by 46% after treatment with celecoxib (p<0.05) when compared with control-treated animals. Serum PGE2 levels were significantly decreased by 30-40% in celecoxib-treated mice fed a high and low fat diet compared with the placebo control groups (p<0.01) (Figure 4D), suggesting that celecoxib suppresses the activity of COX-2 in the KpB mice.

After treatment with celecoxib or placebo, the protein expression of Ki-67, COX-2, cleaved caspase-3 and matrix metalloproteinase-9 (MMP9) in the ovarian tumors was evaluated by immunohistochemistry (IHC) (Figure 4E). The expression of COX-2 was significantly reduced by 60% and 40% in the obese and non-obese groups treated with celecoxib compared with the control treated mice, respectively (p<0.01). Celecoxib inhibited Ki-67 expression, a marker of cell proliferation, in the treated mice on a high fat diet (obese) by 22% and those on a low fat diet (non-obese) by 11%. Celecoxib increased cleaved caspase-3 expression from 18-50% in the tumors from the obese group as compared to 8-22% in the non-obese group, suggesting an enhanced effect of celecoxib on apoptosis in the setting of obesity. MMP9 has been implicated in invasion and metastasis in ovarian cancer. Celecoxib treatment reduced MMP9 expression by 27% in the obese group and by 20% in the non-obese group as compared to controls.

### The effect of celecoxib on angiogenesis in ovarian cancer cells and tumors

**Figure 5:**
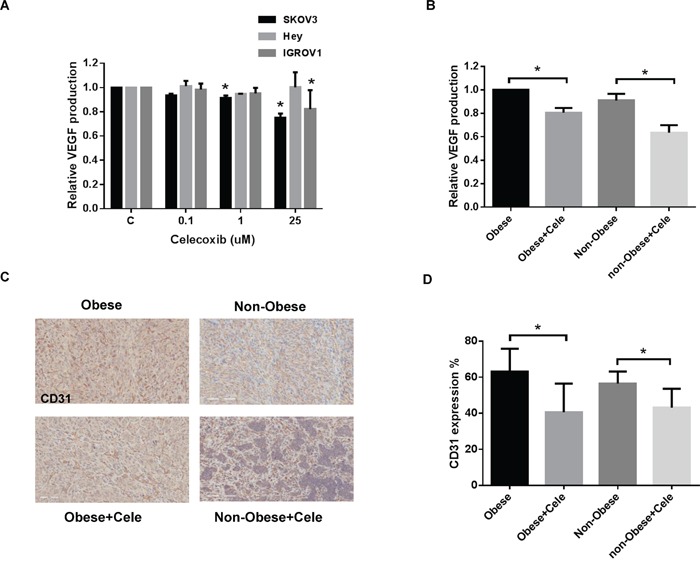
Celecoxib inhibited angiogenesis *in vitro and in vivo* Celecoxib reduced the production of VEGF in the media of SKOV3, Hey and IGROV1 cells **A.** after 24 hours of treatment and mouse serum from obese and non-obese KpB mice **B.** Immunohistochemistry results for CD31 indicated that celecoxib reduced the density of tumor blood vessels in the obese and non-obese KpB ovarian cancer model **C and D.** (*p<0.05).

VEGF is a key mediator of angiogenesis, a process that is important in ovarian cancer growth and metastasis. We investigated the effect of celecoxib in the inhibition of secretory VEGF, a pro-angiogenic factor responsible for the migration and invasion of ovarian cancer cells. VEGF secretion in serum-free culture media was assessed in all three ovarian cancer cell lines by ELISA after 24 hours of treatment. Celecoxib notably decreased VEGF secretion compared with controls in the SKOV3 and IGROV1 cell lines, whereas celecoxib treatment did not show any significant change in the secreted VEGF levels in the Hey cell line (Figure 5A).

Serum levels of VEGF were found to be decreased significantly in the obese and non-obese KpB mice treated with celecoxib as compared to controls (Figure 5B). The level of serum VEGF decreased by 20% in the ovarian tumors from obese mice and 27% in the ovarian tumors from non-obese mice. To further confirm the role of decreased VEGF activity induced by treatment with celecoxib in the formation of blood vessels, we analyzed the treated ovarian tumors through staining with the endothelial cell marker, CD31 (Figure 5C and 5D). Ovarian tumors from the obese and non-obese mice treated with celecoxib had significantly reduced blood vessel density (23% in the obese group and 13% in the non-obese group), in comparison to placebo-treated mice.

### Celecoxib inhibited proliferation of primary cultures of ovarian cancer cells

**Figure 6:**
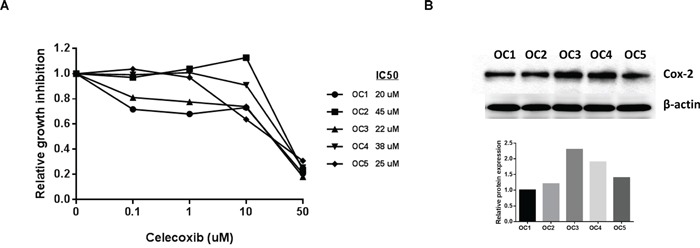
Celecoxib inhibited cell proliferation in primary cell cultures of human ovarian cancer Five primary cell cultures of ovarian cancer were cultured in 96 well plates and treated with celecoxib as indicated doses for 72 hours. Cell proliferation was assessed by MTT assay **A.** COX-2 protein expression did not predict sensitivity to celecoxib in the primary cultures of ovarian cancer **B.** (*p<0.05).

We next investigated the effects of celecoxib on cell growth in human primary cultures of ovarian cancer using the MTT assay. These tissue samples were obtained from patients undergoing surgery for primary ovarian cancer. After 72 hours of treatment, all five primary cultures responded to the celecoxib treatment, showing a significant level of cytotoxicity and inhibition of cell growth at a dose up to 50 uM with a range of IC50 values from 20-45 uM (Figure 6A). In order to determine if the level of COX-2 protein expression was related to the sensitivity to celecoxib in each primary culture case, we assessed COX-2 protein expression using Western blotting in the five untreated primary cell cultures. The results revealed varying levels of COX-2 expression in the five primary cultures (Figure 6B). An analysis of the data using a linear regression model showed the levels of COX-2 protein expression did not correlate to sensitivity to celcecoxib in the five primary ovarian cancer cultures.

## DISCUSSION

Celecoxib has been shown to have anti-tumorigenic effects in various types of human cancer cells and animal models [21]. Treatment with celecoxib has been shown to act on multiple targets and pathways in cancer cells, including proliferation, apoptosis, angiogenesis, invasion and tumor-induced immune suppression *via* COX-2 dependent and independent mechanisms [21, 25, 26]. COX-2 has been found to promote resistance to apoptosis and enhanced survival by regulating protein expression of NF-kB, SERCA, PDK-1/Akt, survivin, Bcl-2 and Mcl-1 [26–29]. It is thought that COX-2 inhibitors interfere with tumorigenesis and apoptosis through decreased PEG2 production, a prostaglandin product of COX-2 [29, 30]. High levels of COX-2 have been found to promote resistance to apoptosis by altering the relative protein levels of survivin and of the Bcl-2 family [30]. Conversely, decreased levels of COX-2 protein expression and PGE2 as the result of celecoxib treatment have been correlated with increases in apoptosis in cancer cells [30]. These findings suggest that celecoxib is not simply blocking the function of COX-2 but may also be disrupting COX-2 protein production [30]. Further support of this is that the anti-apoptotic effects of COX-2 are better correlated to decreases in COX-2 protein expression than inhibition of enzymatic activity [26, 30].

We found that celecoxib treatment significantly reduced COX-2 protein expression and PGE2 production in ovarian cancer cell lines, primary cultures of ovarian cancer and the KpB ovarian cancer mouse model. Celecoxib decreased cell growth, increased cleaved caspase-3 activity and induced cell cycle G1 phase arrest in a dose-dependent manner in three ovarian cancer cell lines. At 72 hours of treatment with celecoxib, the IC50 for SKOV3, IGROV1 and Hey cells was 25, 44 and 50 uM, respectively, and five primary cultures of ovarian cancer had achievable IC50 values (between 20-45 uM). hTERT, a marker of proliferating cells [31, 32], was statistically decreased in all ovarian cancer cell lines treated with celecoxib. In addition, celecoxib inhibited ovarian tumor growth, induced apoptosis and reduced angiogenesis in obese and non-obese KpB mice. Our results are in agreement with those previously reported in ovarian cancer cell lines and xenograft models [27, 33, 34], suggesting that celecoxib may have promise as a potential treatment for ovarian cancer.

Invasion and metastasis are the leading causes for recurrence, poor prognosis and death in ovarian cancer patients [35, 36]. Adhesion and invasion are early steps involved in the metastatic process for ovarian cancer, which has a complex molecular basis that likely involves adhesion molecules, cell surface receptors, oncogenes, chloride channels, fatty acid synthase and focal adhesion kinase [37, 38] Overexpression of COX-2 leads to phenotypic changes in cancer cells that may enhance their tumorigenic potential and invasive ability [39]. PGE2 production also favors the migration of cancer cells to endothelial cells [40]. Several studies have reported that celecoxib inhibits adhesion and invasion in gastric cancer, oral cancer, lung cancer, colon cancer and osteosarcoma cells through multiple cell signaling pathways such as NF-kB, MMP-2/9, E-cadherin, β-catenin and Akt/PKB [41–43]. Among these, matrix metalloproteinases (MMPs) plays an important role in invasion and metastasis for a number of malignancies[44]. Overexpression of COX-2 in lung cancer cells, through transfection with a COX-2 expression vector, has been shown to result in increased protein expression of MMPs and E-cadherin as well as corresponding enhanced invasion and migration abilities [45, 46]. Inhibition of COX-2 expression by celecoxib leads to decreased secretion of MMP-2 and MMP-9 and suppression of the metastatic potential in animal models [46]. In addition, treatment with PGE2 has been found to partially enhance cell migration and invasion in various types of human cancer cells [47]. We found that celecoxib significantly inhibited adhesion and invasion in all three ovarian cancer cells tested and reduced MMP9 protein expression, PGE2 production and blood vessel density in the ovarian tumors from obese and non-obese KpB ovarian mice, suggesting that celecoxib has both anti-tumorigenic and anti-metastatic effects in ovarian cancer.

Accumulating evidence suggests that obesity is a significant risk factor for ovarian cancer and is associated with worse outcomes for this disease [4–18]. Obesity leads to elevated expression of insulin, estrogen, growth factors, inflammatory cytokines and adipokines, which promote ovarian cancer cell proliferation, survival, metastasis, angiogenesis and reduced apoptosis in cancer cells. Obesity-associated inflammation is thought to be one of the most important factors connecting obesity to cancer [19, 48, 49]. Multiple lines of preclinical evidence demonstrate that COX-2 contributes to obesity and obesity-induced muscular insulin resistance [48]. COX-2–derived PGE2 has been shown to up-regulate aromatase through a cAMP–protein kinase A (PKA) cascade in human breast tissue, and the production of COX-2–derived PGE2 in the breast tissue has been positively correlated with both obesity and breast inflammation in women [50]. Thus, these potential benefits of COX-2 inhibition provide a rationale for celecoxib's use in ovarian treatment and prevention, specifically for high risk obese women with this disease [51, 52].

Although celecoxib has been shown to inhibit tumor growth in several animal cancer models, including ovarian cancer xenograft-bearing mice [27, 34], less is known of celecoxib's impact on ovarian tumor growth in genetically engineered mouse models or in obese *versus* non-obese mice. In this study, we mimicked a clinically obese state of ovarian cancer by using the KpB ovarian cancer model and by feeding these mice either a LFD or HFD once the mice reached 3 weeks of age. Celecoxib inhibited the growth of ovarian cancer in obese and non-obese KpB mice without any noticeable toxicity. Treatment with celecoxib in the obese group appeared to have greater anti-tumor efficacy than in the LFD group when comparing tumor volume and tumor weight between the two groups (66% *versus* 46%, respectively) (Figure 5A–5C). Inhibition of ovarian tumor growth in both obese and non-obese mice was significantly associated with decreased levels of COX-2, MMP9 and Ki-67 protein expression, reduction of blood vessel density and increased apoptosis. Although the mechanisms by which celecoxib inhibits the growth of the tumor *in vivo* are currently unclear, our data suggests that celecoxib exerts its anti-tumorigenic effects in the KpB ovarian cancer mouse model *via* a COX-2-dependent pathway through induction of apoptosis, inhibition of tumor angiogenesis and reduction of the cell proliferation index.

Ovarian cancer growth is angiogenesis-dependent, and VEGF is the most potent and specific angiogenic growth factor. Overexpression of VEGF plays a pivotal role in angiogenesis, carcinogenesis and progression of ovarian cancer [53]. COX-2 has been found to stimulate angiogenesis by promoting the production of VEGF and by increasing products of PGE2 and prostacycline [54]. Overexpression of VEGF in primary tumor and serum is associated with poor progression-free survival (PFS) and overall survival (OS) for patients with ovarian cancer [54, 55]. Celecoxib strongly decreased the serum level of VEGF in the KpB ovarian cancer mouse models under obese and non-obese conditions. Treatment with celecoxib resulted in a dose-dependent inhibition of the production of VEGF in cell culture media in human ovarian cancer cells. In addition, we observed a statistically significant decrease in blood vessel density in each treatment group, as shown by determining the CD31 protein expression in the ovarian tumors from the KpB mice. Further review of the mouse ovarian cancer tumors showed large areas of central necrosis in celecoxib-treated tumors, consistent with an insufficient blood supply (data not shown). The decrease in tumor-associated production of VEGF and blood vessel density by celecoxib could be one of the major mechanisms by which it reduces angiogenesis and inhibits overall ovarian cancer growth in the KpB mouse model

**Figure 7:**
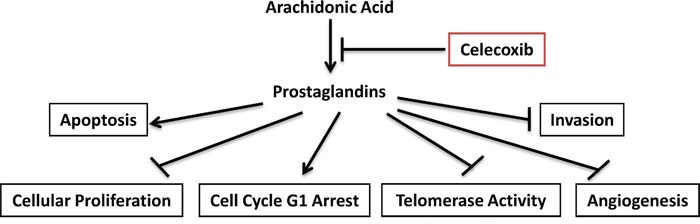
Schematic presentation of the action of celecoxib in ovarian cancer Celecoxib inhibited COX-2 activity and reduced products of prostaglandins, finally resulted in inhibition of cell proliferation and hTERT RNA expression, induction of apoptosis and cell cycle G1 arrest, reduction of cell invasion and in ovarian cancer.

In conclusion, we found that the celecoxib is a potent inhibitor of ovarian cancer cell growth *in vitro* and *in vivo* under different metabolic conditions (obese *versus* non-obese) through different targets (Figure 7). Celecoxib is currently being evaluated in clinical trials as a potential chemotherapeutic and chemopreventive agent for a variety of different cancers, including colon, lung, breast and ovarian cancer [20, 25, 26, 56, 57]. Celecoxib in combination with carboplatin was well-tolerated and found to have a promising response rate of 28.9% in a heavily pre-treated group of women with recurrent ovarian cancer [56]. In addition, there has been a randomized, phase 2 clinical trial of celecoxib plus docetaxl/carboplatin as first line treatment in ovarian cancer patients [57]. In this clinical trial, the addition of celecoxib did not seem to improve PFS or OS; however, 24% of the patients on celecoxib stopped this drug early due to skin reactions which may have undermined the potential benefit of this treatment [57]. Thus, further experimental and clinical studies are warranted to fully assess the potential benefit of celecoxib alone or in combination with other common therapeutic agents in women with ovarian cancer.

## MATERIALS AND METHODS

### Cell culture and reagents

Three ovarian cancer cell lines, SKOV3, Hey and IGROV1, were used for these experiments. SKOV3 cells were grown in DMEM/F12 media which was supplemented with 10% bovine, 100 units/ml penicillin and 100 ug/ml streptomycin under 5% CO_2_. The Hey and IGROV1 cells were grown in RPMI 1640 supplemented with 5% and 10% fetal bovine serum, 300 mM l-glutamine, 10,000 U/ml penicillin and 10,000 μg/ml streptomycin under 5% CO_2_. Celecoxib was obtained from Pfizer (Groton, CT). MTT, RNase A and RIPA buffer were obtained from Sigma (St. Louis, MO). Cleaved caspase 3 activity assay kit was bought from AAT Bioquest (Sunnyvale, CA). The ChemoTx invasion kit was from NeuroProbe (Gaithersburg, MD). Enhanced chemiluminescence Western immunoblotting detection reagents were obtained from Amersham (Arlington Heights, IL). All other chemicals were obtained from Sigma (St. Louis, MO).

### Cell proliferation assays

The SKOV3, Hey and IGROV1 cells were plated in 96-well plates at a concentration of 5000 cells/well for 24 hours. These cells were then treated with various concentrations of celecoxib for a period of 24-72 hours. After the addition of MTT dye (5 mg/mL), the 96-well plates were incubated for 1-2 hours at 37°C. 100 uL of DMSO was added to the plates in order to terminate the MTT reaction, and the plates were read by measuring absorption at 595 nm. The effect of celecoxib was calculated as a percentage of control cell growth obtained from PBS (1%) treated cells grown in the same 96-well plates. Each experiment was repeated three times to assess for consistency of results.

### Flow cytometry

The three ovarian cancer cell lines were plated at 2.5-3.5 × 10^5^ cells/well in 6-well plates in the appropriate media for 24 hours. The cells were then treated with celecoxib at varying concentrations for 24 hours. After treatment, the cells were washed twice with PBS, fixed in 90% methanol solution and were then stored in a −20°C freezer until analysis. The cells were washed twice with cold PBS, centrifuged, suspended in 100 uL PBS and 10 uL of RNase A solution (250 ug/mL) and then incubated for 30 minutes at 37°C. After incubation, 110 uL of PI (100 ug/mL) stain was added to each tube and incubated for 30 minutes at 4°C. Flow cytometric analysis was conducted using a CyAn flow cytometer (Beckman Coulter, Miami, FL). ModFit (Verity Software House, Topsham, ME) was utilized for the analysis to control for dead cells and debris. The experiments were performed in triplicate and repeated twice to assess for consistency of response.

### Caspase-3 activity assay

Cleaved caspase-3 activity was assessed using cleaved caspase-3 activity assay kit. After treating the cells with celecoxib in 96-well plates, 10 ul of caspase-3 assay loading buffer was added into each well and mixed gently. The plates were then incubated for 60 min at 37°C, 5% CO_2_. The fluorescence intensity was measured at an excitation wavelength of 350 nm and an emission wavelength of 450 nm using a plate reader from Tecan (Morrisville, NC). Each experiment was repeated at least twice for consistency of response.

### Real-time RT-PCR for hTERT

Analysis of the effect of celecoxib on hTERT gene expression was conducted using real time RT-PCR. Total RNA was extracted from the three ovarian cancer cell lines using the RNAqueous kit (Ambion, Austin, TX) and further purified by the DNA-free kit (Ambion). The reverse transcription and PCR reactions were performed using the TaqMan Gold one step RT-PCR kit in the ABI Prism 7700 Sequence Detection System (Applied Biosystems, Foster City, CA). Reverse transcription was carried out at 48°C for 30 minutes. The PCR conditions consisted of a 10 minute step at 95°C, 40 cycles at 95°C for 15 seconds each and 1 minute at 65°C. The housekeeping control gene, acidic ribosomal phosphoprotein P0 (RPLP0), was used as an internal control to correct for differences in the amount of RNA in each sample. The standard curve for hTERT was generated by using dilutions of a known amount of cRNA synthesized by *in vitro* transcription of a cloned fragment. The normalized level of hTERT in each sample was estimated by a ratio of the hTERT level to the RPLP0 level [31]. Each experiment was performed in triplicate and repeated twice to assess for consistency of results.

### Western immunoblotting

The SKOV3, Hey and IGROV1 cells were plated at 2-4 × 10^5^ cells/well in 6-well plates in their appropriate media and were treated for 24 hours with celecoxib. Cell lysates were prepared in RIPA buffer (1% NP40, 0.5 sodium deoxycholate and 0.1% SDS) plus PhosStop. Equal amounts of protein were separated by gel electrophoresis and transferred onto a PVDF membrane. The membrane was blocked with 5% nonfat dry milk and then incubated with a 1:1000 dilution of primary antibody overnight at 4°C. The membrane was then washed and incubated with a secondary peroxidase conjugated antibody for 1 hour. Antibody binding was detected using an enhanced chemiluminescence detection buffer and the Alpha Innotech imaging system (San Leandro, CA). After developing, the membrane was re-probed using an antibody against α-tubulin or β-actin as a control. Each experiment was repeated three times to assess for consistency of results.

### Prostaglandin E2 (PGE2) assay

The ovarian cancer cell lines were plated in 24 well plates and cultured for 24 hours. The cells were then treated with celecoxib for 18 hours. The culture media was harvested using a syringe and filtered through a 0.22-mm filter (Millipore, Billerica, MA). PGE2 productions in the media was determined by quantitative enzyme-linked immunosorbent assay (ELISA) (Cayman Chemical, Ann Arbor, MI), according to the manufacturer's instructions. PGE2 concentration was measured in triplicate for each cell line. The concentration of PGE2 in the serum of mice treated with celecoxib was also assessed by this technique.

### Adhesion assay

96-well plates were used, and each well was coated with 100 uL laminin-1 and incubated at 37°C for 1 hour. The coating was then aspirated, and 200 uL of blocking buffer was added to each well and incubated for 45-60 minutes at 37°C. PBS was used to wash the wells and the plates were chilled on ice. To each well, 2.5 × 10^3^ cells were added with PBS and varying concentrations of celecoxib, and the plates were incubated at 37°C for 2 hours. The media was then aspirated, and the cells were fixed by adding 100 uL of 5% glutaraldehyde and incubating for 30 minutes at room temperature. Adhered cells were washed with PBS and stained with 100 uL of 10% of crystal violet for 10-30 minutes. The cells were then washed repeatedly with sterile water, and 100 uL of 10% acetic acid was added to each well to solubilize the dye. After 5 minutes of shaking, the absorbance was measured at 570 nm using a FLUOstar OMEGA plate reader from BMG Labtech (Cary, NC). The experiments were repeated three times to assess for consistency of results.

### Invasion assay

The ChemoTx invasion kit (Gaithersburg, MD) was used to assess invasion in all three ovarian cancer cell lines. The cells were placed in a serum free media for a period of 24 hours. The cells were then collected, washed and re-suspended in Gey's Balanced Salt Solution + 1% BSA, with varying concentrations of celecoxib. To each well, 299 uL of media plus varying concentrations of celecoxib was added, and the framed filter membrane was carefully fitted to the top of the plate. The plate was then incubated for 4 hours at 37°C to allow invasion into the lower compartment. 3 uL of MTT (5 mg/ml) was used to stain these cells at 37°C for one hour. The liquid was aspirated, and the wells washed with PBS. The MTT dye was solubilized using 200 uL of DMSO. The absorbance was measured at 595 nM using a FLUOstar OMEGA plate reader (BMG Labtech, Cary, NC).

### Measurement of VEGF levels

To measure VEGF levels, the SKOV3, Hey and IGROV1 cell lines (2.5×10^5^ cells) were plated in 6-well plates and incubated under standard culture conditions overnight. Subsequently, the media was replaced by serum-free culture media, and varying concentrations of celecoxib were added. The media was collected after 24 hours of exposure to celecoxib. 10-50 ul of culture media was used to measure the levels of VEGF with a VEGF ELISA kit (DVE00, R&D Systems, Minneapolis, MN), according to the manufacturer's instructions. The optical density at 570 nm of each well was measured using a FLUOstar OMEGA reader (Cary, NC). The VEGF concentration in the serum of mice after exposure to celecoxib was also measured by the same VEGF ELISA kit.

### Obesity and the K18-gT_121_^+/−^;p53^fl/fl^; Brca1^fl/fl^ mouse model

The K18-gT_121_^+/−^;p53^fl/fl^;Brca1^fl/fl^ (KpB) mouse model (Terry Van Dyke, PhD, NCI) is a serous OC mouse model, wherein the tumor suppressor genes, Brca1 and p53 are specifically and somatically deleted and the retinoblastoma (Rb) proteins are inactivated in the adult ovarian surface epithelium [58]. Inactivation of all 3 Rb proteins by T_121_ (a fragment of the SV40 large T antigen) is driven by the keratin 18 (K18) promoter [58]. Expression of the T_121_ transgene and knockout of p53 and Brca1 are conditional and only activated *via* injection of an adenoviral vector expressing Cre (AdCre) into the ovarian bursa cavity of adult female mice. At approximately 6 months after AdCre injection, tumors develop in the affected ovary, while the un-injected ovary remains normal.

All experimental animals were maintained in accordance with the Institutional Animal Care and Use Committee (IACUC) and the NIH guide for the Care and Use of Laboratory Animals. Recombinant adenovirus Ad5-CMV-Cre (AdCre) was purchased from the University of Iowa Transfer Vector Core at a titer of 10^11^−10^12^ infectious particles/ml. To maximize weight gain, mice were provided a high-fat diet (HFD, obese group) (60% kcal from fat, Research Diets, New Brunswick, NJ) and control mice (non-obese group) were provided a low-fat diet (LFD) (10% kcal from fat, Research Diets, New Brunswick, NJ) *ad libitum*, beginning at 3 weeks of age [59]. AdCre injection occurred at 6 weeks to induce OC [58]. Thirty-six hours following superovulation, the mice were anesthetized, and a single 1 cm incision was made on the dorsal surface of each mouse. The AdCre was then injected *via* a needle introduced into the oviduct near the infundibulum and into the ovarian bursa, and the incision was closed.

The KpB mice were monitored weekly by palpation for tumor growth. Celecoxib and placebo treatment was initiated after palpation of a 1 cm tumor in mice on the HFD (obese group) and LFD (non-obese group) (N = 15 mice per group). Celecoxib was dissolved in DMSO at 5 mg/ml, further diluted 10 times in 0.5% methylcellulose with 0.025% Tween 80 and injected (IP) daily at a dose of 5 mg/kg body weight for 4 weeks. The tumor sizes were measured once a week by palpation. Tumor volume was calculated using the following equation: volume (mm3) = a × b2/2, where is the largest diameter and b is the smallest diameter. The animals were weighed weekly throughout the study. At sacrifice, mice were weighed and blood samples were taken. Half of the ovarian tumor was snap-frozen and stored at −80°C, and the other half was fixed in 10% neutral-buffered formalin and paraffin embedded. Mouse heart, lungs and kidneys were also harvested, fixed in formalin and grossly examined for any suspicious lesions before paraffin embedding.

### Immunohistochemistry

Five micron paraffin sections of the ovarian tumors from the KpB mice were used for immunohistochemical analysis. Staining procedures were performed with the assistance of the Immunohistochemical Mouse Core Facility at the University of North Carolina at Chapel Hill. The following primary antibodies were used: Ki-67 (Cell Signaling, Beverly, MA, 1:800), MMP-9 and CD31 (Santa Cruz, Santa Cruz, CA, 1:500 and 1:100), cleaved caspase-3 (Cell Signaling, Beverly, MA, 1:100) and COX-2 (Cell Signaling, Beverly, MA, 1:300). Further processing was carried out using ABC-Staining Kits (Vector Labs, Burlingame, CA) and hematoxylin. Immunochemistry slides were scanned, analyzed and scored by Aperio and related ImageScope software (Vista, CA).

### Primary culture of human ovarian cancer cells

Five human ovarian tumor specimens were obtained from patients undergoing surgery for serous ovarian carcinoma at the University of North Carolina at Chapel Hill. The protocol was reviewed and exemption granted by the Institutional Review Board at the University of North Carolina at Chapel Hill. For the culture of primary ovarian cancer cells, the freshly obtained tissues were washed three times with Hank's Buffered Salt Solution (HBSS), and then gently minced by scissors in DMEM/F12 media containing 10% fetal bovine serum (FBS). These tissues were then digested in 0.2% collagenase IA, 100 U/ml penicillin and anti-anti for 30-60 min hours at 37°C water bath with shaking. After two centrifugations with PBS solution, cells were re-suspended and diluted to 1×10^5^ cells/ml with DMEM/F12 media. 1×10^4^ cells/well were then seeded into 96-well plates and incubated for 24 hours before celecoxib treatment. Cell proliferation was measured with MTT assay 72 hours after treatment.

### Statistical analysis

All of the experiments were repeated a minimum of three times. Data were presented as mean ± S.E.M. Statistical analyses of the differences between the groups were determined with the two-sided unpaired Student's t-test using GraphPad software (La Jolla, CA USA), and a value of p<0.05 was considered as significant.
